# Exploring the Engaged Worker over Time—A Week-Level Study of How Positive and Negative Work Events Affect Work Engagement

**DOI:** 10.3390/ijerph18136699

**Published:** 2021-06-22

**Authors:** Oliver Weigelt, Antje Schmitt, Christine J. Syrek, Sandra Ohly

**Affiliations:** 1 Institute of Psychology–Wilhelm Wundt, Leipzig University, D-04109 Leipzig, Germany; 2 Work and Organizational Psychology, University of Hagen, D-58084 Hagen, Germany; 3 Organizational Psychology, University of Groningen, 9712 TS Groningen, The Netherlands; a.schmitt@rug.nl; 4 Business Psychology, University of Applied Sciences Bonn-Rhein-Sieg, Von-Liebig-Str. 20, D-53359 Rheinbach, Germany; christine.syrek@h-brs.de; 5 Business Psychology, University of Kassel, D-34121 Kassel, Germany; ohly@uni-kassel.de

**Keywords:** affective events, work engagement, sensitization-satiation effects, job demands-resources model, experience sampling, growth curve modeling

## Abstract

Although work events can be regarded as pivotal elements of organizational life, only a few studies have examined how positive and negative events relate to and combine to affect work engagement over time. Theory suggests that, to better understand how current events affect work engagement (WE), we have to account for recent events that have preceded these current events. We present competing theoretical views on how recent and current work events may affect employees (e.g., getting used to a high frequency of negative events or becoming more sensitive to negative events). Although the occurrence of events implies discrete changes in the experience of work, prior research has not considered whether work events actually accumulate to sustained mid-term changes in WE. To address these gaps in the literature, we conducted a week-level longitudinal study across a period of 15 consecutive weeks among 135 employees, which yielded 849 weekly observations. While positive events were associated with higher levels of WE within the same week, negative events were not. Our results support neither satiation nor sensitization processes. However, a high frequency of negative events in the preceding week amplified the beneficial effects of positive events on WE in the current week. Growth curve analyses show that the benefits of positive events accumulate to sustain high levels of WE. WE dissipates in the absence of a continuous experience of positive events. Our study adds a temporal component by highlighting that positive events affect work engagement, particularly in light of recent negative events. Our study informs research that has taken a feature-oriented perspective on the dynamic interplay of job demands and resources.

## 1. Introduction

From a psychological perspective, organizational life can be understood in terms of a chain of events [1]. Despite calls to take issues of time more seriously [2,3,4,5], researchers in the field of occupational health psychology have only recently begun to consider the dynamics of relevant phenomena, such as employee strain and engagement [6], through the lens of work events [7]. Work engagement has been described as “a positive, fulfilling, work-related state of mind that is characterized by vigor, dedication, and absorption” [8]. Furthermore, work engagement has attracted considerable research interest within the last 15 years [9,10,11]. In particular, job characteristics have been identified as major drivers of work engagement [6,10,11], and empirical evidence has consistently shown that work engagement is determined by the interplay of different types of job characteristics (e.g., resources such as autonomy and demands such as workload) [12]. However, to fully understand the experience of work and how it relates to employee outcomes (e.g., engagement), it is advisable to go beyond generalized perceptions of how a job usually is (i.e., job characteristics measured by surveys in terms of job demands and resources). More specifically, there is a need to consider dynamic aspects (i.e., day-to-day fluctuations in job characteristics) [12,13] as well as factors that are more proximal to employee experiences over time [14]. Therefore, a focus on enacted job characteristics—that is, events and activities in the job as they happen [14]—is warranted. Work events differ from job features in that they are “discrete and bounded in space and time“ [1]. Therefore, the study of work events (versus job characteristics) would provide the opportunity to add a temporal component to the research on job characteristics [6] and to examine work events as more proximal antecedents of work engagement [13,14]. In other words, the study of work events rather than job characteristics would provide the opportunity to specify and examine how the different situations that employees experience at work combine to affect work engagement over time. For instance, over the course of a workweek, employees are likely to experience a series of positive events (e.g., praise from a supervisor after successfully finishing an important task) and negative events (e.g., an episode of interpersonal conflict with colleagues). Although the occurrence of each of these events is individually associated with short-term fluctuations in work engagement in its own right [15], it is likely that the last week’s work events carry over to affect work engagement during the current workweek [16]. Furthermore, different work events may interact to predict work engagement. Put another way, receiving praise from one’s supervisor in the current week’s team meeting may foster work engagement, but the events of the previous week (e.g., positive feedback from the same supervisor or interpersonal conflict with colleagues) may alter the impact of this current event [see 1]. Hence, it is worthwhile to consider work events embedded within a chain of events over time [17]. To account for the richness of the work experience [18], we drew on a taxonomy of work events that encompasses a broad range of relevant positive and negative work events [7]. This taxonomy was derived from qualitative research [7] and can be considered exhaustive with regard to the most relevant work events from the perspective of employees. The taxonomy provides an integrative framework covering a broad range of work events that have been considered in the literature to date (see [7] for a literature review). In the present study, we leverage this taxonomy to determine which specific type of work event is most relevant to work engagement, aside from the effects of positive and negative work events in general terms. 

Above, we have outlined that an event-oriented approach permits specifying the order of what happens and interactions among current events and recent events. Recently, Wickham and Knee [16] have proposed applying experience sampling data to analyze such interactions between current events and recent events to describe psychological processes of sensitization and satiation over time. For instance, in the case of sensitization, last week’s conflict makes the current week’s conflict seem worse. That is, employees become more vulnerable or susceptible to work events with each episode. Conversely, in the case of satiation, last week’s conflict makes this week’s conflict seem less threatening. In other words, employees become less vulnerable or susceptible to work events with each episode. We adopt this approach to examine sensitization and satiation to the study of both positive and negative events predicting work engagement. Furthermore, a positive event like praise from the supervisor may yield particularly strong effects on work engagement after a negative event has occurred [17,19,20]. Hence, we extend the sensitization-satiation perspective and scrutinize the interplay of positive events and negative events from one week to the next week. Interestingly, experiencing a set of events in a given order (e.g., conflict with colleagues after praise from the supervisor) may not be equivalent to the reverse order and is likely to result in different levels of work engagement. However, theory and empirical research on job characteristics and work engagement so far have largely focused on the situational features of work [12] and have rarely considered temporal issues in depth. Put another way, research on job demands and resources usually does not distinguish between experiencing a specific resource prior to or after being confronted with high levels of a specific job demand. Accordingly, in this study, we aim to account for the order of positive and negative events and examine competing hypotheses. Given that job characteristics are linked to work events as more proximal precursors of work engagement [13,14], our event-oriented temporal approach has implications beyond the study of work events per se. In this sense, the different types of work events correspond to immediate situational consequences of a broad range of job characteristics [13,14]. Hence, our research informs researchers interested in the interplay of job demands and job resources and may contribute to reconcile inconsistent findings on this interplay as well.

On a related note, it is important to gain insights into how frequent exposure to positive and negative events may accumulate to affect work engagement over longer periods of time [6,21,22]. These insights are important as they pave the way to connect transient processes to longer-term processes underlying employee well-being [21]. In the study of work events, researchers have rarely gone beyond considering the cross-sectional associations or short-term effects of events over a couple of hours (see [7] for a review). Hence, we know little about sustained effects due to the accumulation of negative or positive events over time. However, if work events do not have longer-term implications for individual outcomes, one may question their practical relevance [21]. Conversely, studying accumulation effects may contribute to gain insights in how mundane events in the daily grind of work add up and lead to potentially profound changes in work engagement over time. We therefore conducted a week-level diary study over a period of four months, which fits these aims best: capturing meaningful events shortly after they happen, but, at the same time, monitoring mid-term changes in work engagement by applying an intensified longitudinal design.

Our study contributes to the literature in at least two ways. First, we add a temporal perspective to the research on relationships between job characteristics and work engagement by considering sensitization and satiation to positive and negative events. This approach is important because it allows us to specify the order of events and the interplay of current and recent events. Second, we extend the sensitization-satiation perspective and examine whether positive and negative events combine to affect work engagement from one week to the next. In this sense, we follow the call for applying experience sampling data for analyzing the effects of work events within the context of a history of preceding events [1,15].

### 1.1. What Happens in the Short Run: Work Events as Antecedents of Work Engagement

**Figure 1:**
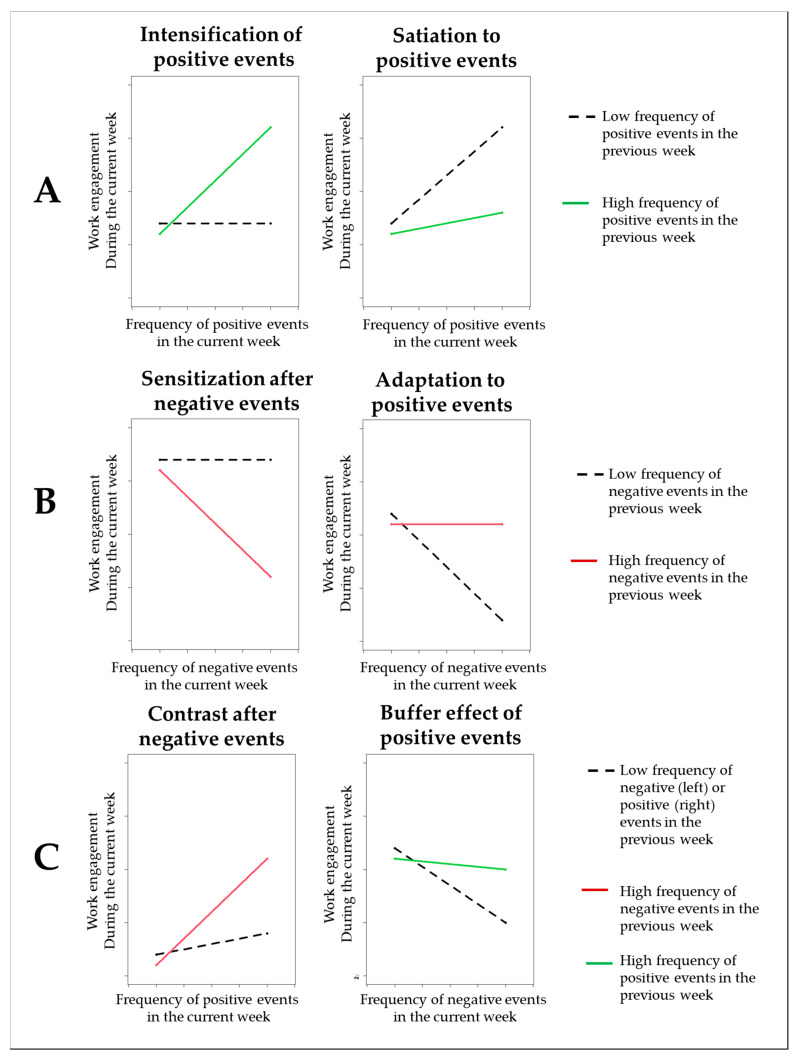
Prototypical ways of how work events may interact to predict work engagement. (Panel **A**) refers to the interplay of positive events over time. (Panel **B**) refers to the interplay of negative events over time. (Panel **C**) refers to the interplay of positive and negative events over time.

In recent years, evidence on antecedents of work engagement at the intraindividual level has started to accumulate [12]. However, links between work events and work engagement have rarely been considered explicitly. According to Weiss and Cropanzano [13], affective events are “things [that] happen to people in work settings” to which “people react emotionally” (p. 11). From the perspective of the conservation of resources theory [23], positive events signal the availability of resources or opportunities for resource gain [24]. Given that positive work events refer to experiences that either overlap in content with or are triggered by resources such as rewards or reinforcement [12,25,26], we assume that positive events at work are positively related to work engagement. Accordingly, positive work events, such as praise from supervisors, predict work engagement within [19,27] and between individuals [26]. By contrast, negative events can be considered factors that detract attention and may inhibit engagement in focal tasks [28]. So far, empirical evidence on negative events and work engagement has been mixed. One study has favored significant negative links between negative events and work engagement at the day-level [15,19]. By contrast, other researchers found negligible lagged associations with work engagement [29]. Their results suggest no lagged main effects of previous-day positive event intensity on work engagement the next day. Moreover, in some studies negative events paradoxically even yielded beneficial lagged effects on job satisfaction [17] and work engagement [19]. More specifically, given these inconsistencies, we need to account for what happens in the aftermath of the focal events. Events probably do not affect employee well-being in isolation, and it is unlikely that “participants in diary studies … become a tabula rasa once they have completed the diary report for a given interval” [16]. Therefore, the present study incorporates a temporal component and considers work events embedded in a series of events that may happen to an employee over time [1,17]. For one, we take into account that the effects of recent events on work engagement may carry over from one week to the next and affect work engagement. Second, we consider how past events affect the impact of current work events. Given that there are contrasting views on what the interplay of work events may look like, we formulate competing hypotheses. Prototypical patterns of interactions are depicted in Figure 1. Panel A refers to prototypical patterns of work engagement that may arise from the interaction of current × lagged positive events. Panel B describes prototypical patterns for interactions of current × lagged negative events. Finally, Panel C illustrates how positive events and negative events may combine over time to affect work engagement. Given that we aim to extend the perspective beyond prior-day-level research, we focus on links and interactions at the week-level, which is a time frame rarely applied to work events. This approach appears to be adequate, because the seven-day week is a salient unit for structuring time [30]. Furthermore, week-to-week associations tap into less transient and more profound effects over time [31].

### 1.2. Temporal Patterns of Positive Events

While the concurrent association between positive events and work engagement is well-established [15,19,26,27,29], the carryover effects of positive events on work engagement have rarely been considered (see [29] for the only exception). However, [29] was focused on negative event intensity and several features of their design (e.g., events sampled on three consecutive days only, time frame of focal measures referred to the day level), their measures (e.g., affective reaction to events versus frequency of events as predictor), and their focal analyses (e.g., coefficients for positive events when controlling for several other aspects) prevent us from drawing strong conclusions regarding lagged effects of positive events per se. Basically, there are two perspectives: First, positive events experienced in the course of the previous workweek may linger on to affect work engagement in the current week, for instance by means of positive reflection (e.g., about successfully finishing a project) [32] or capitalization on the same event through social sharing with others [33]. Second, positive events from the previous workweek may change the way current positive events are perceived and experienced. To investigate these temporal processes, Wickham and Knee [16] have suggested applying interactions of current events (concurrent) and more recent events (lagged) to experience sampling data. As illustrated in Figure 1A, there are two prototypical patterns of the interaction. On the one hand, employees may get used to high frequencies of positive events. For instance, research on the hedonic treadmill suggests that individual standards may change, and positive events will be taken for granted when positive events have occurred frequently before [34]. That is, in light of many positive events in the previous week, current-week high frequencies of positive events have a reduced impact on work engagement. Throughout this manuscript, we label this pattern the satiation effect (right side of Figure 1A) (Wickham and Knee [15]). On the other hand, positive events in the past may contribute to benefits even more from current positive events, as positive events broaden awareness for positive events that might follow [35]. Throughout this manuscript, we label this pattern the intensification effect (right side of Figure 1A). Positive events may even trigger behaviors of the individual that provoke positive events in the future [36]. Given that there are competing theoretical views and that prior empirical results do not allow for firm conclusions, we state two competing hypotheses for satiation versus intensification effects:

**Hypothesis** **1.** *Concurrent positive events in week n and lagged positive events in week n-1 interact to predict work engagement in week n. Lagged positive events (a) amplify (intensification) or (b) alleviate the effect of concurrent positive events (satiation).*

### 1.3. Temporal Patterns of Negative Events

The rationale regarding sensitization and satiation effects presented above can also be applied to negative events. The prototypical patterns of work engagement are illustrated in Figure 1B. Negative work events in the previous workweek may affect employees even after a couple of days have passed [29,37]. So, negative events in the current workweek may shift attention to negative cues in the environment and make employees react more sensitively to negative events during the next workweek [22,38]. In line with this perspective, Farmer and Kashdan [39] found that individuals reacted more sensitively to negative social events on a given day when negative events had preceded the day before. A prototypical pattern of work engagement is depicted on the left side of Figure 1B. Throughout this manuscript, we label this pattern the sensitization effect.

By contrast, from the perspective of the allostatic load model [40], it is also plausible that employees will adapt to negative events and will not mind negative events when they re-occur. This may be because employees might become more proficient in dealing with negative events [41] or become more resilient due to having been challenged before [42]. Throughout this manuscript, we label this pattern adaptation [43]. In sum, negative events in the previous workweek, may make employees either more susceptible to the detrimental effects of negative events (sensitization) or may contribute to adapting to negative events (adaptation, see right side of Figure 1B). Again, we state two competing hypotheses:

**Hypothesis** **2.** *Concurrent negative events in week n and lagged negative events in week n-1 interact to predict work engagement in week n. Lagged negative events (a) amplify (sensitization) or (b) reduce the effect of concurrent negative events (adaptation).*

### 1.4. The Interplay of Positive and Negative Events over Time

Beyond sensitization and satiation effects, our study addresses the question of whether experiencing positive events in the aftermath of negative events results in different levels of work engagement than experiencing positive events after a period of few positive events. Above we have discussed that positive events in the previous week may broaden awareness of and strengthen the impact of current positive events. In a similar way, negative events in the past may also change the way current positive events are perceived. For instance, experience-sampling research on work events and after-work fatigue—a state of tiredness and reduced functional capacity—suggest that employees benefit most from positive events in the face of negative events and in the face of chronically high job demands [20]. Other researchers have argued that work engagement results from a shift in affect in the aftermath of negative events – that is, down-regulation of negative affect and up-regulation of positive affect [19]. Empirically, these authors found that negative events enhance, rather than impair work engagement, when followed by high levels of positive affect. Given that prior research is mute on the triggers of affective shift and the beneficial effects on work engagement, we consider positive work events to be predictors, because positive events have consistently been linked to positive affective outcomes [7]. Positive events in the aftermath of negative events may be particularly beneficial for work engagement because positive events create a contrast effect at the backdrop of prior negative events [20,44,45]. Accordingly, and in line with the contrast after a negative-events perspective, we expect that negative events in the past and current positive events interact to predict work engagement. A prototypical pattern of work engagement is depicted on the left side of Figure 1C. Throughout this manuscript, we label this pattern the contrast effect. More specifically, we expect that positive events in the aftermath of negative events will have a particularly strong effect on work engagement:

**Hypothesis** **3.** *Concurrent positive events in week n and lagged negative events in week n-1 interact to predict work engagement in week n. Lagged negative events amplify the effect of positive events (contrast effect after negative events).*

To gain a more complete picture of how positive and negative events interact over time, we need to consider whether positive events in the past change the impact of current negative events. We argue, that positive events in the previous workweek may also contribute to build up personal resources [24,25] that change the way current negative events affect work engagement. For instance, a high frequency of positive events in the previous workweek is associated with positive affect [7] and may therefore replenish coping resources [46]. In this sense, positive events likely strengthen self-efficacy [47] and self-regulation capacity [48] as personal resources (see, for instance [49]). Hence, after experiencing positive events in the previous workweek, employees may be well-equipped to face negative events in the current week. In line with this idea, Kuba and Scheibe [15] found that habitual acceptance as a personal resource buffers the detrimental effects of negative events on work engagement at the day-level. Given that positive events likely feed personal resources and that resources, in turn, attenuate the detrimental effects of negative events on work engagement, we assume that positive events in the previous workweek attenuate the impact of negative events in the current week. Throughout this manuscript we label this pattern the buffering effect. A prototypical pattern of work engagement is depicted on the right side of Figure 1C.

**Hypothesis** **4.** *Concurrent negative events in week n and lagged positive events in week n-1 interact to predict work engagement in week n. Lagged positive events attenuate the effect of current negative events (buffering effect).*

### 1.5. What Happens in the Long Run: Sustained Effects of Work Events over Time

Recently, Ilies and Aw (2015) have reviewed the theory and empirical evidence on intraindividual models of well-being and noted that we need to connect transient processes (as reflected in fluctuations in well-being from day to day) to longer-term processes (as reflected in changes in well-being over periods of weeks, months, or years). If applied research provides evidence that, for instance, positive events are associated with sustained changes in work engagement over longer periods of time, these findings would underscore the practical relevance of these concepts in organizations from a practitioner’s point of view, whereas associations at the day- or week-level may reflect fluctuations around characteristic average levels that might be largely stable over time (see also [34]), sustained effects address the issue of whether work events indeed yield chronically beneficial effects [21]. Given that prior intraindividual research has not considered this aspect empirically, we examine whether frequent exposure to positive and negative events is associated with mid-term changes in work engagement over time at the interindividual level.

Drawing on the conservation of resources theory [23], it has been suggested that work engagement results from resource abundance [50,51]. According to Halbesleben and colleagues [25], positive aspects in organizational settings like social support, justice, and trust act as signals that the “investment of resources will help the individual realize his or her goal of achieving more resources.” (p. 1347). Given that positive events tap into these kinds of signals, we assume that a high frequency of such signals over time is associated with gains in work engagement. The frequent experience of positive events over time should accumulate to feed higher levels of work engagement. In other words, trajectories of work engagement should be more positive (steeper increase) when positive events occur frequently compared to when positive events occur infrequently.

**Hypothesis** **5.** *Trajectories in work engagement differ between persons dependent upon the frequency of positive events over time. Higher (lower) frequencies of positive events are associated with steeper (flatter) increases in work engagement.*

Given the pioneering nature of our study with regard to mid-term trajectories of work engagement dependent upon accumulation of work events, we do not state a formal hypothesis on the effects the frequency of negative events over time might have. However, we do investigate the concurrent effects of the frequency of negative events within our focal analyses on the accumulation of positive events. Our analyses, therefore, also provide insights into the relative importance of positive versus negative events for work engagement in the long run.

## 2. Materials and Methods 

### 2.1. Procedure

Drawing on the rationale outlined above, we conducted a week-level diary study across a period of four months. Participants filled in a general survey containing demographics and other variables assumed to be largely stable across time. After registering for the study and filling out the general survey, participants received emails inviting them to complete short diary questionnaires across a period of 15 consecutive weeks with two questionnaires per week. The procedure and materials of this study have not undergone examination by an ethics committee, as the measures and procedures of our study followed the protocols of standard self-report experience sampling research in applied psychology, and we did not touch sensitive topics (e.g. sexual orientation). Our study fully complied with the standards of the Department of Psychology at the University of Hagen, which included strict guidelines to guarantee the anonymity of the self-reported data. Individuals interested in participating in our study were informed about the general aims and the protocol of the study before their participation. All participants gave their informed consent for inclusion before they participated in the study. The study was conducted in accordance with the Declaration of Helsinki. Our protocol did not include any form of deception of participants. Participation was voluntary, and participants had the opportunity to quit whenever they wanted.

### 2.2. Sample

Our 135 participants were employees who were enrolled in a psychology distance-learning program at a German university that offers these courses primarily for individuals who study besides their regular jobs and occupations. Participants received course credit for filling out the general survey and the diary questionnaires. Credit was commensurate with the number of completed weekly surveys and participants who completed ten or more surveys received some extra credit.

Seventy-seven percent of our participants were female. The average age was 35.41 years (SD = 9.93), ranging from 18 to 61 years. Tenure within the organization ranged from less than one year to 28 years (M = 6.79, SD = 7.34). Participants came from diverse industries, mainly from healthcare (19%), the service sector (16%), education (10%), and commerce (9%). Participants had either full-time or part-time jobs and worked, on average, 32.18 h per week (SD = 9.92); 75% had a permanent contract, and 29% had a leadership position. In total, we received 849 observations (person-weeks) for Friday from 135 persons (on average 6.3 weeks per person, 42% of the theoretically possible 2025 observations) suited for use in our growth curve models. Our analyses of short-term lagged effects from one week to the next week, however, relied on matched observations from two consecutive weeks. Given that participants had missing data for single or several weeks over the course of 15 weeks, our analyses of the short-term effects were based on a sample of 490 matched observations from 101 employees. Descriptive information and zero-order correlations for the full sample and the matched sample at the intraindividual level and at the interindividual level are presented in Table 1 and Table 2, respectively.

### 2.3. Measures

We applied short versions of validated scales adapted to the purposes of our study. Participants rated aspects on 5-point Likert scales to indicate the frequency of experiences during the recent workweek. Unless stated otherwise, response options ranged from 1 (“never during this week”) to 5 (“several times a day”).

#### 2.3.1. Positive Work and Negative Work Events during the Workweek

We measured work events within the recent workweek on Friday afternoon using eleven items from the work-events checklist, which covers the work-events clusters identified by Ohly and Schmitt [7]. The work-event checklist consists of 13 items, two of which refer to events not directly related to the job (negative events: bad news in employees’ private lives and health problems). Given the focus and theoretical rationale of the present study, we confined analyses to a set of eleven items, which were explicitly job-related. However, we included the off-job events in the supplemental analyses. Five items tapped into positive events during the current workweek. Sample items are “Did you get confronted with positive but unexpected news or information (e.g., a promotion or a new work order)?” and “Did you receive a positive feedback or a thank from anyone (e.g., supervisor, colleagues or customers)?” We applied six items to capture negative events within the recent workweek. Sample items are, “Did you experience any conflicts or communication problems with colleagues?” and “Did you experience a situation that negatively affected the working climate and the cooperation among the employees/colleagues in your department/your company (e.g., dismissal of a colleague, issues dealing with the supervisor, unsuccessful team meetings)?” Multilevel McDonald’s Omega for positive events is 0.69 at the intraindividual level and 0.83 at the interindividual level. Multilevel McDonald’s Omega for negative events is 0.58 at the intraindividual level and 0.73 at the interindividual level. Although reliability is important for measures in general, it is less so with regard to factual information such as the one we assessed ((How often) Did the event occur?). Given that work events are formative rather than reflective constructs coefficient alphas or omegas are not optimal for judging reliability [52]. For instance, experiencing high levels of conflict does not necessarily imply high levels of ambiguity or organizational changes at the same time. Keeping this in mind, the reliability of the work-events measures seems to be adequate.

#### 2.3.2. Work Engagement during the Workweek

We applied a brief three-item measure to capture work engagement based on the UWES-9 items (Utrecht Work Engagement Scale) [53]. Preliminary analyses based on cross-sectional data from the baseline survey of the present study using the UWES-9 items (Utrecht Work Engagement Scale) [53] suggested that all items loaded on one factor (see [54] on the structure of the UWES) and, in our study, the three highest loading items captured engagement as reliable as the UWES-9 in our baseline survey (r UWES9-UWES3 = 0.97). We applied the following items: “During this week, I was enthusiastic about my job” (dedication dimension); “During this week, I was immersed in my work” (absorption dimension); and “During this week, I got carried away when I was working” (absorption dimension). We calculated multilevel alphas for work engagement following procedures introduced by [55], implemented in R by [56]. Alphas for work engagement were 0.84 at the intraindividual level and 0.96 at the interindividual level. Given that alpha has been criticized for several reasons, we report MacDonald’s Omega as an alternative measure of reliability [57]. Omegas were 0.84 and 0.96 at the intraindividual level and at the interindividual level, respectively. Applying the factor analytic procedure outlined below, we found that the three items yielded standardized loadings ranging from 0.75 to 0.82 at the intraindividual level and 0.89 to 0.99 at the interindividual level. The consistently high to very high factor loadings provide evidence that our measure of work engagement is reliable and taps into a single construct.

### 2.4. Analytic Strategy

We examined the reliabilities of the focal measures by means of multilevel confirmatory factor analysis applying the lavaan library in R [58]. We estimated models applying the robust maximum likelihood estimator (MLR), which is recommended for non-normally distributed data [59]. We applied the semTools library in R [60] to estimate multilevel reliabilities inferred from the focal confirmatory factor analysis models.

In focal analyses, we applied multilevel modeling [61] to account for the dependence of repeated observations. We applied the “nlme” package for R [62]. As weekly observations were nested within persons, we specified two-level models. Work engagement yielded an intraclass correlation coefficient (ICC(1)) of 0.61. In our focal analyses, predictors at the week-level (Level-1) were centered around the person-mean [63]. Given that we expected relationships between predictors and criteria to vary across persons, we specified random effects for all focal predictors. We controlled for the Level 2-effects of our focal predictors [16,64,65] and entered the person-means of positive and negative events for each person to predict the intercept of work engagement. The person-mean of positive or negative events captures the amount of work events experienced over the period of 15 weeks. Including the person-mean of positive and negative work events at Level 2 offers the advantage of being able to differentiate between differences at the interindividual level and the focal short-term effects at the intraindividual level [66]. Our model is equivalent to what Kreft et al. call a CWC_2_ model [67]. That is, a model applying predictors centered within context (CWC) and including the cluster-mean (2).

To analyze the mid-term effects of frequent exposure to work events over time, we specified growth curve models using multilevel modeling. We followed the steps recommended by [68] for growth curve modeling using a multilevel modeling approach in R. We specified linear changes (decrease or increase) in work engagement over time as a random slope of time in weeks predicting these outcomes. Significant random effects indicate that employees differ in the rate of change in the respective outcome variable. We also probed quadratic and cubic trajectories for exploratory purposes. We then added the person-means of positive and negative work events as cross-level moderators, which tests whether differences in the trajectory of work engagement (slope of time) can be explained by the amount of positive and negative events experienced by each person over time. Whereas the person-means as covariates depict differences in characteristic average levels of work engagement due to frequent exposure to work events, the trajectories can be interpreted as increases or decreases in weekly work engagement over time.

## 3. Results

In a first step, we examined whether each type of positive and negative work events had occurred or not (once or several times versus not at all during the workweek) and how frequently these events had occurred over the course of the 15 weeks. With regard to positive events we found that positive events occurred more frequently than negative events. Positive events ranged from more than 335 occurrences (work-related good news) to more than 828 occurrences (goal attainment, problem-solving, and task-related success). Negative events ranged from more than 327 occurrences (problems in interactions with clients) to more than 460 occurrences (ambiguity, insecurity, and loss of control). Average frequencies for each type of event are displayed in Table 1 for descriptive purposes. Whereas positive events occurred on average several times a week, negative events occurred on average less than once a week during the period studied.

In the second step, we ran multilevel factor analyses to examine the reliability of the focal measures. We specified a three-factor model (positive events, negative events, work engagement) homologous across levels of analysis. That is, we specified a three-factor model at the intraindividual level and a three-factor model at the interindividual level [66]. We compared the model fit of a three-factor model to alternative models, such as a single-factor model and a two-factor model with work events and engagement loading on distinct factors. We found that the three-factor model achieved an acceptable fit as reflected in Comparative Fit Index (CFI) = 0.945, Tucker-Lewis Index (TLI) = 0.932, Root Mean Square Error of Approximation (RMSEA) = 0.035, and Standardized Root Mean Square Residual (SRMR) _within_ = 0.053 and SRMR _between_ = 0.081. The three-factor model fit the data better than the alternative models (Delta χ² > 144.55, *p* < 0.001).

### 3.1. Short-Term Effects of Work Events

Addressing the first set of hypotheses, we specified Model 1, in which work engagement (in week *n*) was regressed on the main effects of concurrent (week *n*) and lagged work events (week *n*−1), the interactions among positive events (satiation or intensification) and among negative events (adaptation or sensitization). We found that models, including auto-regressive and heteroscedasticity specification, did not improve model fit [68] and did not alter the pattern of results. Therefore, we omitted these specifications from the focal models. Results are depicted in Table 3. We found a positive relationship between positive events during the workweek and work engagement in the same week (γ = 0.74, t = 12.52, *p* < 0.001) at the intraindividual level. Concurrent negative events were unrelated to work engagement (γ = 0.07, t = 0.86, *p* > 0.10). We did not find evidence for the lagged main effects of work events from week n−1 to week n. That is, neither positive nor negative events carried over to affect work engagement from one week to the next. Furthermore, concurrent positive events did not interact with lagged positive events (γ = −0.07, t = −0.61, *p* > 0.10). Hence, in contrast to Hypothesis 1, we found neither sensitization nor satiation effects of positive events. In a similar way, concurrent negative events did not significantly interact with lagged negative events to predict work engagement (γ = −0.07, t = −0.07, *p* > 0.10). Hence, in contrast to Hypothesis 2, we found neither sensitization nor satiation effects of negative events. Repeated exposure to positive events does not change the way positive events affect work engagement in the next week. The same holds for negative events.

**Figure 2:**
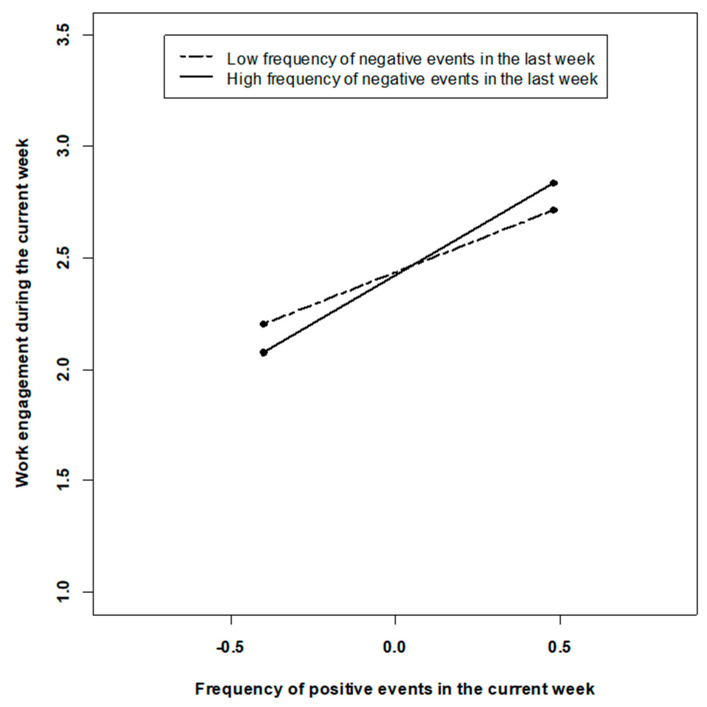
Interaction of current positive and lagged negative events at the week-level.

Addressing Hypotheses 3 (contrast after negative events) and 4 (buffering effect), we examined the interactions of lagged negative events x current positive events and of lagged positive events x current negative events. In line with Hypothesis 3, we found that lagged negative events and concurrent positive events interact to predict work engagement (γ = −39, t = 2.36, *p* = 0.008). The pattern of the interaction is depicted in Figure 2 and suggests that frequent negative events in the last week amplify the positive association between positive events and work engagement in the current week (Simple slopes: γ low negative events = 0.60, t = 7.70, *p* < 0.001, γ high negative events = 0.86, t = 11.41, *p* < 0.001). Gains in work engagement at the week-level due to positive events are greatest in weeks when many negative events have preceded in the week before. In contrast to Hypothesis 4, lagged positive events did not change the effects of concurrent negative events (γ = 0.11, t = 0.67, *p* > 0.10). In sum, our results are compatible with the basic idea of a contrast effect after negative events. However, we did not find evidence for sensitization or satiation effects across weeks.

### 3.2. Mid-Term Changes in Work Engagement Due to Work Events

**Figure 3:**
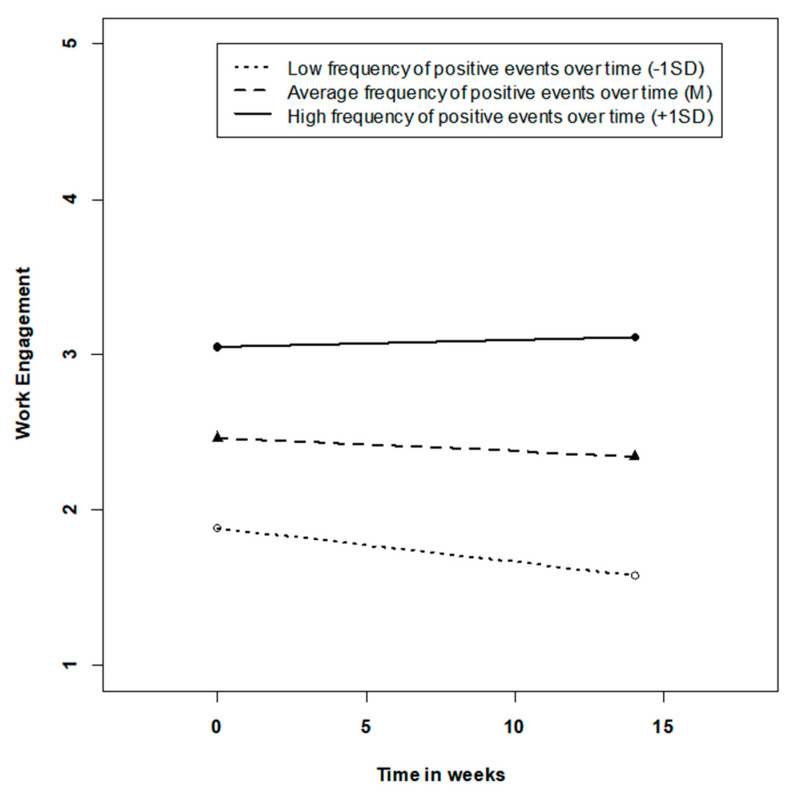
Trajectories of work engagement over 15 weeks dependent upon the accumulation of positive events over time.

Results from linear growth curve models predicting changes in work engagement over time are shown in Table 4 (Growth Model 1). In a first step, we found a significant negative effect of time (γ = −0.01, t = −2.05, *p* = 0.04), indicating that, on average, work engagement slightly decreased over the period of four months. Given that we found significant slope variance, we considered the frequency of positive and negative events over time as cross-level moderators in Growth Model 2. In line with Hypothesis 5, positive events were predictive of the slope of time (γ = 0.03, t = 1.97, *p* = 0.04). In contrast, negative events did not contribute to explain slopes in work engagement over time (γ = −0.02, t = −1.13, *p* > 0.10). The trajectories of work engagement over time dependent upon accumulation of positive events are depicted in Figure 3. Inspection of the slopes reveals that lower frequencies of positive events over time are related to steeper decreases in work engagement over time, whereas work engagement remains constant when a high frequency of positive events occurs. Further inspection of simple slopes using tools developed by [69] suggests that work engagement decreases when the frequency of positive events over time is close to the grand-mean or below and that work engagement might even increase when very high frequencies of positive events are present (region of significance −0.01 > w > 1.57) (simple slopes: γ low positive events = −0.03, t = 2.97, *p* < 0.01, γ high positive events = −0.00, t = 0.04, *p* > 0.10). Besides the trajectories over time, the person-mean of positive events was also predictive of the intercept (γ = 0.98, t = 8.36, *p* < 0.001). That is, differences in individual “characteristic average levels” [21] of work engagement were attributable to the frequency of positive events over time. Work engagement was higher for individuals who experienced positive events more frequently over the period of four months.

### 3.3. Additional Analyses

We ran several additional analyses to scrutinize the robustness of our results, address potential alternative explanations, and explore additional issues related to the link between work events and work engagement. First, to rule out systematic bias due to missing data, we reran Models 1–4 using subsamples of participants, who had provided either at least 8 (*n* = 51) or 10 (*n* = 39) out of 15 weekly reports. The pattern of results did not differ from those of our focal analyses. That is, all main and interactions effects remained significant. These findings suggest that the number of missing observations did not systematically affect the focal results, implying that the focal effects are robust. Models using a subsample of participants who provided at least 12 reports per person yielded convergence problems in Model 2 due to the low number of participants (*n* = 20) and fall below the threshold for minimum sample sizes at Level 2. Detailed results of the supplemental analyses will be provided by the first author upon request.

Second, in our focal analyses, we have combined different types of positive events to a global measure of positive events and then applied the same strategy to negative events. This approach serves to draw comparisons with prior research that has distinguished between positive versus negative events in general terms. However, in the present study, we applied an 11-item work-events checklist that also included two items referring to off-the-job events, namely health-related problems and negative news in employees’ private lives.

This allows for a more fine-grained analysis of the relative strength of the association between work events and work engagement. Whereas prior research only indicates that positive events tend to be beneficial for work engagement, the present study aimed to determine which types of events may be most relevant for work engagement at the week-level and hence, which classes of events are actual drivers of work engagement. Following a similar analytic strategy as in prior research on the comprehensive work-events taxonomy [7], we ran multilevel models and regressed work engagement at the week-level on all types of work events. We applied the full sample for these analyses and specified random intercepts and fixed slopes for each type of work event because the sample sizes at both levels of analysis did not permit specifying eleven random slopes within the same model. The results are displayed in Table 5. In essence, we found almost all types of positive work events uniquely contribute to explain the variance in week-level work engagement. More specifically, goal attainment events (γ = 0.23, t = 6.69, *p* < 0.001); passively experienced positive events (γ = 0.16, t = 5.59, *p* < 0.001); and episodes of praise, appreciation, and positive feedback (γ = 0.20, t = 6.45, *p* < 0.001) were positively related to levels of work engagement. Furthermore, perceived competence through social interactions was significantly related to higher levels of work engagement at the week-level too (γ = 0.07, t = 1.97, *p* = 0.049), albeit the coefficient was a bit lower than for other work events. By contrast, negative events were unrelated to week-level work engagement, except for episodes of ambiguity, insecurity, and loss of control. Interestingly, the coefficient for this type of negative work event was positive rather than negative (γ = 0.07, t = 2.11, *p* = 0.034. Hence, this type of negative event contributes to enhance, rather than diminish, work engagement, when considered in concert with all other types of work events. As the other negative work events, negative off-job events did not yield significant associations with work engagement. The consistent evidence across all types of positive (negative) events supports our approach of forming composite scores for positive and negative events, respectively.

Third, our study provides the opportunity to assess whether associations between positive work events and work engagement within the same week are due to common method bias only. More specifically, we leveraged the matched sample and ran an alternative version of Model 2 regressing work engagement in week n on positive and negative work events in week *n*, lagged positive and negative work events in week n−1 controlling for work engagement in week n-1. In other words, we controlled for prior levels of the outcome variable when predicting week-level work engagement. Finding significant associations between our focal predictors and work engagement under these circumstances would facilitate the interpretation of results as work events predicting changes in work engagement rather than both phenomena co-occurring at the same time. The results are presented in Table 6. In essence, we found the same pattern of results as in our focal analyses. That is, the main effect of positive work events at Level 1 (γ = 0.72, t = 12.19, *p* < 0.001) and the interaction at Level 1 remained significant (γ = 0.37, t = 2.43, *p* = 0.015). Not surprisingly, previous week’s work engagement was positively linked to current week’s work engagement (γ = 0.27, t = 6.29, *p* < 0.001). Of note however, the inclusion of work engagement from the previous week resulted in a significant lagged effect of positive events in week n-1 on work engagement in week n (γ = -.19, t = –2.85, *p* = 0.005).

Fourth, we probed whether positive and negative events interact *within the same week* to predict work engagement. This perspective would be in line with the perspective of prior research on work events that has not accounted for the order of events (e.g., [20]). Moreover, this kind of concurrent work-events interaction corresponds to the perspective taken in experience-sampling research on job demands and resources. We specified an alternative version of Model 2 including the interaction of positive x negative events within the same week. In essence, when analyzing the full sample, we found evidence for a positive link between positive events and work engagement at the week-level (γ = 0.69, *t* = 13.35, *p* < 0.001) and that positive and negative events interact to predict work engagement (γ = −0.31, *t* = –2.76, *p* = 0.006). An inspection of the simple slopes confirmed that negative events alleviate the link of positive events and work engagement within the same week. However, an analysis of concurrent interactions across two consecutive weeks in the matched sample (including all combinations of positive events, negative events, lagged positive events, and lagged negative events) revealed no interactions of concurrent positive and negative events within the same week (γ = −13, *t* = −0.83, *p* = 0.41). The results of these supplemental analyses suggest that the pattern of interaction of concurrent positive and negative events is opposite to the pattern of interaction when taking into account the order of events. Negative events alleviate the link between positive events when measured concurrently with positive events. However, negative events amplify the link between positive events and work engagement when measured prior to positive events.

Finally, we complemented our growth modeling analyses and examined whether positive and negative events accumulate in the long run to explain interindividual differences in work engagement. To infer accumulation, we estimated so-called emergent or compositional effects leveraging multilevel modeling procedures [61,67,70,71]. In the case of intensified longitudinal data, emergent effects refer to between-person differences in the outcome variable that can be attributed to between-person differences in the predictor variable over time. We estimated the emergent effects of positive (negative) events by subtracting the within-person from the between-persons effect, that is, holding constant the momentary weekly level of positive (negative) events, respectively. The emergent effect reflects the expected difference in work engagement between two persons who have the same frequency of positive (negative) events in a given week but who differ by one unit of the overall level of positive (negative) events. We expected that even if two persons report similar levels of positive (negative) events in a particular week, their work engagement might differ depending on the frequency of work events experienced over four months. We found a positive emergent effect of positive events (γ = 0.38, t = 3.67, *p* < 0.001) and a negative emergent effect of negative events (γ = -.64, t = −4.01, *p* < 0.001). That is, experiencing positive events more frequently over time is associated with higher levels of work engagement. Experiencing negative events more frequently over time is associated with lower levels of work engagement.

## 4. Discussion

In this study, we have examined how positive and negative events dynamically interact to predict fluctuations in work engagement from week to week. Notably, we have added a temporal component [19], which might resolve inconsistent findings in prior research. Furthermore, our study is among the first to explicitly consider whether the accumulation of work events is predictive of the mid-term trajectories of work engagement over a period of four months. Our approach complements prior research on job demands and resources as more distal feature-oriented antecedents of work engagement, such as time pressure or autonomy [12,72], and provides a more nuanced picture of the interplay of positive and negative events over time.

First, our results extend prior research, which has reported that negative events may, under certain circumstances, be beneficial for work engagement, dependent upon what happens afterwards [19]. The present study contributes to clarify the dynamics underlying these seemingly paradoxical effects [17]. Specifically, our results suggest that the occurrence of positive events is tightly related to high levels of work engagement and that current positive events affect work engagement particularly in the light of recent negative events. High levels of work engagement result from a contrast that evolves when experiencing positive events in the aftermath of negative events. The amplifying effect of recent negative events on the association between current positive events and work engagement is consistent with research on the affective-shift model of work engagement [19] and is also in line with the interplay of job demands and job resources as postulated in job demands-resources theory [6]. However, taking into account the order of positive and negative events provides a more differentiated picture. Whereas recent negative events interacted with current positive events, recent positive events did not interact with current negative events to predict work engagement. So, the timing of positive and negative events may play a crucial role. In this sense, our results illustrate the value of studying the experience of work through the lens of work events and taking the order of events into account. Our results suggest that, for instance, experiencing support after struggling with overload results in different levels of work engagement than facing overload in the aftermath of support. In feature-oriented research on job demands and resources, researchers usually do not account for this distinction. The results suggest that we need to consider these temporal aspects to avoid inconsistent results in the future. Our supplemental analyses show that, while negative events alleviate the link between positive events and week-level work engagement, negative events amplify the link between positive events and work engagement, when negative events precede positive events. In this sense, our study may help explain why interactions of demands and resources have emerged in some studies but have not been found in other studies applying feature-oriented approaches to the interplay of job characteristics measured concurrently. One reason for these inconsistencies may be that measures applied in feature-oriented research neglect the temporal order of relevant events and result in mixed findings, depending on which timeframe employees have in mind when thinking about time pressure, organizational constraints, perceived progress towards goal attainment, or praise from their supervisor.

Given that we did not find sensitization or satiation effects either for positive or for negative events, obviously, gains in work engagement do not result from a contrast between currently low frequencies of negative events versus high frequencies of negative events in the previous week (adaptation). In the same way, the positive events of the previous week do not alter the impact of this week’s positive events on work engagement (intensification), but negative events of the previous week do. Importantly, whereas positive events yielded strong direct short-term associations with work engagement, negative events merely acted as the background for positive events, which amplifies the gains due to positive events—a pattern similar to the effects of positive events on fatigue in the face of high job demands [20]. Furthermore, our analysis of lagged effects from one week to the next suggests that work events apparently do not directly carry over from the previous week to the next week. Associations of positive and negative events with work engagement found in prior day-level research [15,19], therefore, seem to reflect short-lived effects, which fade out rather quickly within a couple of hours [3]. Admittedly, our measures of work events were focused on mundane, rather than exceptional, work events and therefore, may underestimate how long the beneficial or detrimental effects may actually last. The impact of work events varies as a function of event strength and event duration [1,73]. For instance, the impact of novel or highly disruptive events like psychological contract breach [37] may not fade out after a couple of hours or days but will likely take longer [1]. Our supplemental analyses on the unique links of work events with work engagement within the same week suggest that almost all types of positive events quite consistently covary with work engagement.

Second, we rigorously tested whether work events yield sustained—and hence, practically meaningful—significant changes in employee engagement [21]. More specifically, our approach taps into accumulation effects over time. Given that knowledge about accumulation effects and the timing of both positive and negative events is scarce, our results add to current theoretical perspectives [1,73]. We found that, on average, work engagement tends to decrease, and frequent exposure to positive events over time is associated with slower rates of change over time or constantly high levels of work engagement. For a high frequency of positive events, a flat linear trend results, a pattern described as a “passageway trajectory” in the literature (cf. [25]). The general downward trend is in line with the notion that work is associated with investment and thereby, the consumption of resources over time. Our results are in line with research that has provided evidence for “some downward pressure on the general upward trend” [25]. This downward trend is also consistent with declining trajectories in variables related to work engagement. For instance, the organizational socialization [74,75] and voluntary turnover literature [76] literature suggests that there may be slow declining trajectories after being very enthusiastic as a newcomer, for instance, due to the accumulation of minor events. Interestingly, our results imply that this downward trend may be compensated for by a high frequency of positive events. By contrast, in our study, negative events did not accumulate to affect work engagement over time. This finding has important implications for understanding the role of positive events for building and sustaining high levels of engagement. Sustained high levels of work engagement over time are dependent upon being fed by frequent positive experiences. In the absence of continuous reinforcement [25], work engagement is likely to fade and decline quite substantially within the daily grind. In this sense, particularly positive events can be considered key drivers to maintaining and fostering engagement.

### 4.1. Practical Implications

From a practical perspective, our findings suggest that single mundane work events have short-term effects on employee work engagement. Positive events have the potential to foster work engagement, and this effect is more pronounced in the aftermath of negative events. However, the frequent occurrence of mundane positive events accumulates to sustain the level of work engagement over periods of several weeks or months. According to our results, in the face of adversity, creating opportunities for positive events afterwards is superior to avoiding the occurrence of additional negative events. Supervisors might acknowledge their followers’ progress towards goal accomplishment as an element of routine communication [77] to foster positive events. Our suggestion coincides with facets of transactional leadership, such as contingent reward and proactive forms of management by exception [78], and stresses the importance of these leadership behaviors in daily job routine. In more general terms, organizations might develop structures and routines that facilitate positive events at work to happen. For instance, adequate job design [79] and optimal employee training are likely to contribute to experiencing successful task completion and positive feedback from others. Beyond goal attainment and successful mastery of job tasks, team meetings have a high potential to act as opportunities for positive social exchange that might feed work engagement (see [7]).

### 4.2. Strengths and Limitations

The key strength of the present study is that we applied an intensified longitudinal design over a period of four months and rigorous methods for analyzing data. A series of robustness checks and supplemental analyses were conducted to qualify our core results. However, the present study relied on self-reported data only. Furthermore the week-level design implied that retrospective reports referred to overall assessments of the entire workweek, an approach that may come at the cost of retrospective bias [80]. On the other hand, we aimed to extend the analysis beyond very short day-level periods, because we intended to capture the impact of rare but potentially powerful events, [29] and we intended to link transient processes to longer-term processes [21]. For instance, quits by colleagues or significant positive team events (e.g., an informal gathering for the celebration of a colleague’s birthday) do not usually occur within a few days but may be important aspects of organizational life [1]; such events are likely to be overlooked in episodic or day-level studies. The relatively low prevalence of negative events of less than one occurrence of each type of negative event per week on average (see Table 1) suggests that the mid-term time-frame of several weeks to months is in line with the relatively rare occurrence of work events that are strong enough to yield sustainable effects over time. Moreover, the results of our supplemental analyses suggest that the associations between positive work events and work engagement within the same week are not purely a result of method-variance [81]. The results held when we controlled for prior levels of work engagement. Although, researchers have recently suggested that affective events may be the results of affective experiences, rather than the other way around [82], the idea of work events affecting affective states, such as work engagement, is consistent with the basic tenets of affective events theory [13]. The results of the present study are compatible with this more traditional view.

Because responses were anonymous, participation was voluntary, and respondents held a wide variety of jobs, we think that our sampling strategy is largely comparable to other psychological studies. However, our sample is not representative of the general working population, so it is unclear how the study findings generalize to other samples. Moreover, we have a high percentage of missing data. We obtained weekly reports for roughly half of the theoretically possible number of observations. This limitation is due to the high number of repeated observations within our ambitious design (fifteen diary surveys in total), which covered a period of almost four months. However, our random coefficient modeling approach does not hinge on listwise deletion and is able to handle missing data. On average, each participant still provided more than six (and nearly five lagged) observations covering periods of at least two months. Furthermore, our supplemental analyses suggest that our results are not dependent upon the number of missing observations. Taken together, we believe that our results are valid despite the missing data. 

Although, our study is among the first to study events based on the work events taxonomy by Ohly and Schmitt [7], we have not distinguished between different clusters of work events (e.g., goal attainment versus praise or perceived competence) in our focal analyses. Consequently, rather than doing a fine-grained analysis of interactions among the five specific positive and six specific negative work-related event clusters identified, our study is meant to provide insights on the general patterns of how positive and negative events (irrespective of their specific content) interact to predict work engagement (for a similar approach, see [19]).

On a related note, several authors [1,73] have argued that the strength of events varies as a function of novelty, disruption, and criticality and should be considered to understand the impact of particular events with regard to individual level or organizational level outcomes. We did not monitor and incorporate these kinds of event characteristics in this study as our focus was on the dynamic interplay of the quantity of work events over time. However, we consider our study a first step towards a better understanding of the dynamics of work events *per se*.

### 4.3. Implications for Future Research

Although our study has addressed several gaps in the literature, a couple of unresolved issues remain to be considered in future research. While some researchers have found negative events to predict lower levels of work engagement [14] (see also Table 3 in [18]), we did not find that negative events had any direct or lagged effects, on average. This inconsistency may have been due to differences in the ways work events were measured (open answer format versus event checklist) or differences in the time lags applied (day-level versus week-level) [3]. For instance, Bone and colleagues [24] provided evidence that the impact of work events on employee health may differ quite substantially depending upon whether data are analyzed at the episodic or at the day-level. Accordingly, it may not be straightforward to generalize results from day-level research to longer time frames [83]. In the present study, it was not clear whether negative events had no affect on work engagement whatsoever or whether their effects simply faded out before work engagement was assessed at the end of the workweek [3]. Day-level data collected over a period of several weeks would allow researchers to gain a clearer picture of how long it takes until the effects of work events unfold or fade out [84]. A combination of day-level and week-level perspectives would further close the gap between transient processes and the longer-term processes discussed above [21]. As noted above, tracking indicators of event strength alongside event frequency could further reconcile contradictory findings and integrate the research on work events into event system theory [1].

On a related note, work events are discrete by definition and are meant to fundamentally and sustainably change the experience of work. Significant work events may even be triggers of transition processes, rather than predictors of minor short-term fluctuations in work engagement [13]. In this sense, future experience sampling research might apply discontinuous growth modeling approaches to account for the discrete nature of (rare but potentially powerful affective) events [76] and study shifts in work engagement, in addition to day-level fluctuation [85].

Furthermore, Koopmann, et al. [86] found that regulatory focus mediates the short-term effects of positive and negative events on employee strain. Drawing on this research and further advancing discontinuous perspectives, future research may also explore the role of shifts of regulatory focus in work engagement fluctuations. Although we have illustrated the dynamic interplay of positive and negative events using examples of events, which may refer to the same task (e.g., struggling with obstacles in a certain task in one week and successfully finishing the task in the consecutive week), we did not track whether the positive events in one week actually were related to the negative events in the preceding workweek. Future research might scrutinize whether compensatory effects are dependent upon a link between the positive and lagged negative events. Furthermore, personality traits, such as positive affectivity, might influence the relationships between positive and negative events, their interplay, and work engagement [19], which needs to be taken into account in future studies.

We have addressed how the repeated occurrence of positive and negative events might accumulate over weeks to months, although our analysis of sensitization and satiation effects across two consecutive weeks yielded no support for a build-up of such effects in the short run. However, we found that the frequent occurrence of positive events is associated with sustained higher levels of work engagement. By contrast, we found emergent effects of both positive and negative events on work engagement at the interindividual level. That is, reporting more positive (negative) events over time is associated with higher (lower) levels of work engagement, even when controlling for momentary levels of positive (negative) work events. Illies and colleagues [21] have emphasized that it is important to explain how transient processes are linked (and lead) to longer-term consequences in terms of employee well-being. Our results suggest that the occurrence of positive and negative events is associated with meaningful differences in work engagement over time. However, we can neither rule out third variables that might explain this link, nor can we establish causality with the correlational data at hand. Future research may take an episodic approach to accumulation effects [87] and leverage linear and discontinuous growth modeling [88] to study build-up effects in a more rigorous way. Our results suggest that positive and negative events yield practically relevant effects and predict interindividual differences in work engagement on the long run (emergent effect) [70,89,90]. Hence, positive and negative events are probably a good starting point to study accumulation effects with regard to work engagement.

## 5. Conclusions

Our study adds a temporal component to the research of work events and work engagement. More specifically, we provide evidence that recent negative events amplify the beneficial effects of current positive events on work engagement. Hence, studying the experience of work through the lens of work events over time [1] improves our understanding of the contingencies and the dynamic interplay that determine work engagement. Furthermore, this study links transient processes to longer-term processes underlying engagement and shows that positive events accumulate to feed continuously high levels of work engagement over periods of several months. Overall, our study provides insights into how work events combine to affect work engagement over time. Notably, our results on mid-term changes in work engagement underscore the practical relevance of work events for employee well-being. We hope our study contributes to provide insights into the vital worker and will inspire further research on what happens at work through the lens of work events in the future.

## Figures and Tables

**Table 1:** Correlations Among Study Variables at the Intraindividual Level.

Variable		*ICC*	1	2	3	4	5	6	7	8	9	10	11	12	13	14
1.	Goal attainment, problem-solving, task-related success	0.40		**0.60**	**0.28**	**0.39**	**0.45**	−0.06	0.05	**0.07**	−0.04	−0.06	0.01	**0.74**	−0.01	**0.38**
2.	Perceived competence in or through social interactions	0.47	**0.62**		**0.25**	**0.41**	**0.56**	−.02	−0.07	0.03	−0.05	**−0.16**	**−0.12**	**0.77**	**−0.10**	**0.45**
3.	Work-related good news	0.30	**0.28**	**0.23**		**0.27**	**0.36**	−0.03	0.03	0.04	**0.09**	−0.02	−0.02	**0.56**	0.03	**0.23**
4.	Passively experienced positive events	0.46	**0.40**	**0.40**	**0.27**		**0.54**	−0.02	**−0.09**	**−0.07**	**−0.15**	**−0.18**	**−0.25**	**0.76**	**−0.20**	**0.45**
5.	Praise, appreciation, positive feedback	0.44	**0.50**	**0.60**	**0.35**	**0.56**		−0.02	**0.07**	−0.02	0.04	**−0.10**	**−0.09**	**0.82**	−0.02	**0.43**
6.	Technical difficulties, problems with work tools and equipment	0.45	−0.07	0.00	−0.05	0.02	0.00		**0.07**	0.05	**0.18**	**0.26**	**0.15**	−0.04	**0.46**	**−0.13**
7.	Hindrances in goal attainment, obstacles in completing work tasks, overload	0.39	**0.12**	−0.02	0.03	−0.08	**0.13**	0.01		**0.36**	**0.44**	**0.33**	**0.23**	0.00	**0.67**	−0.04
8.	Problems in interactions with clients or patients	0.41	0.05	0.00	0.00	−0.06	−0.03	0.00	**0.39**		**0.20**	**0.30**	**0.22**	0.01	**0.54**	0.04
9.	Ambiguity, insecurity, loss of control	0.45	−0.04	−0.03	**0.10**	**−0.13**	0.08	**0.18**	**0.44**	**0.18**		**0.45**	**0.37**	-0.04	**0.71**	**−0.15**
10.	Conflicts and communication problems	0.43	−0.07	**−0.16**	0.00	**−0.16**	−0.06	**0.27**	**0.35**	**0.37**	**0.43**		**0.51**	**−0.15**	**0.74**	**−0.13**
11.	Managerial and internal problems, organizational climate	0.49	0.05	−**0.10**	0.03	−**0.23**	−0.04	**0.10**	**0.27**	**0.26**	**0.33**	**0.46**		**−0.14**	**0.64**	**−0.15**
12.	Positive events	0.53	**0.75**	**0.77**	**0.54**	**0.76**	**0.84**	−0.02	0.05	−0.02	−0.01	**−0.13**	**−0.10**		**−0.09**	**0.54**
13.	Negative events	0.57	0.02	−0.08	0.03	−**0.17**	0.03	**0.43**	**0.69**	**0.55**	**0.70**	**0.74**	**0.63**	-0.05		−**0.15**
14.	Work engagement	0.61	**0.31**	**0.42**	**0.18**	**0.37**	**0.36**	**−0.12**	−0.04	0.07	**−0.17**	**−0.09**	**−0.11**	**0.45**	**−0.13**	
15.	Work engagement (lagged)	--	**0.42**	**0.48**	**0.24**	**0.46**	**0.42**	**−0.13**	−0.01	**0.09**	−**0.17**	−0.08	−0.08	**0.56**	**−0.11**	**0.67**

Note: Correlations above the diagonal are week-level correlations in the full sample (*k* = 849). Correlations below the diagonal are week-level correlations in the matched sample (*k* = 490). Correlations in bold type are significant at *p* < 0.05. Bold is necessary to mark significant correlations.

**Table 2:** Means, Standard Deviations, and Correlations Among Study Variables at the Interindividual Level.

	Variable	*M*	*SD*	1	2	3	4	5	6	7	8	9	10	11	12	13	14	15	16
1.	Gender ^a, b^	0.77	0.42		-−0.06	0.02	0.03	0.02	0.00	0.09	−0.07	−0.12	−0.05	0.04	−0.01	−0.03	0.04	−0.06	0.02
2.	Age in years ^b^	35.75	10.38	−0.01		−0.06	0.13	0.04	0.06	−0.04	−0.01	0.00	−0.04	−**0.28**	−0.15	−0.14	0.04	−0.16	0.11
3.	Goal attainment, problem-solving, task-related success	3.17	0.87	0.15	−0.04		**0.53**	**0.38**	**0.44**	**0.37**	**−0.13**	**−0.19**	0.07	−0.14	−0.14	−0.10	**0.69**	−0.16	**0.45**
4.	Perceived competence in or through social interactions	3.37	0.86	0.12	0.10	**0.72**		**0.39**	**0.51**	**0.64**	−0.05	−**0.22**	0.09	**−0.19**	**−0.23**	**−0.22**	**0.82**	**−0.21**	**0.48**
5.	Work-related good news	1.53	0.75	−0.03	0.03	**0.41**	**0.40**		**0.41**	**0.49**	−0.05	−**0.17**	0.07	−0.04	−0.05	−0.11	**0.67**	−0.09	**0.50**
6.	Passively experienced positive events	2.59	1.11	0.05	0.07	**0.49**	**0.59**	**0.36**		**0.61**	−0.08	−0.17	0.02	**−0.29**	**−0.23**	**−0.34**	**0.80**	**−0.27**	**0.67**
7.	Praise, appreciation, positive feedback	2.47	1.03	0.05	0.04	**0.45**	**0.68**	**0.51**	**0.72**		−0.12	**−0.22**	−0.02	−0.10	**−0.26**	**−0.20**	**0.83**	**−0.23**	**0.63**
8.	Technical difficulties, problems with work tools and equipment	1.76	0.98	−0.02	−0.07	−0.16	**−0.32**	0.01	−0.18	−0.19		**0.28**	**0.18**	**0.27**	**0.43**	**0.34**	−0.11	**0.61**	**−0.23**
9.	Hindrances in goal attainment, obstacles in completing work tasks, overload	1.85	1.1	−0.10	−0.07	−**0.21**	−**0.29**	−0.10	−**0.24**	−0.14	**0.36**		**0.42**	**0.48**	**0.42**	0.15	**−0.25**	**0.67**	−0.13
10.	Problems in interactions with clients or patients	1.56	0.82	0.00	−0.05	−0.06	−0.14	0.04	−0.19	−0.17	0.16	**0.46**		**0.21**	**0.30**	**0.18**	0.06	**0.53**	−0.06
11.	Ambiguity, insecurity, loss of control	1.9	0.98	−0.03	**−0.27**	−0.15	−**0.29**	0.00	−**0.37**	–0.19	**0.45**	**0.56**	**0.30**		**0.58**	**0.52**	**−0.21**	**0.76**	**−0.22**
12.	Conflicts and communication problems	1.6	0.83	−0.07	−0.18	−0.16	−**0.40**	0.01	**−0.34**	**−0.31**	**0.59**	**0.55**	**0.39**	**0.67**		**0.67**	**−0.25**	**0.83**	**−0.26**
13.	Managerial and internal problems, organizational climate	1.63	0.92	−0.09	−0.15	−0.11	−**0.33**	−0.06	**−0.47**	**−0.28**	**0.38**	**0.28**	**0.24**	**0.65**	**0.65**		**−0.26**	**0.71**	**−0.27**
14.	Positive events	2.63	0.68	0.09	0.05	**0.77**	**0.86**	**0.64**	**0.82**	**0.86**	**−** **0.22**	**−** **0.25**	−0.14	**−** **0.27**	**−** **0.32**	**−** **0.33**		**−0.26**	**0.72**
15.	Negative events	1.72	0.59	−0.08	−0.19	**−** **0.20**	**−** **0.40**	−0.03	**−** **0.41**	**−** **0.29**	**0.68**	**0.72**	**0.55**	**0.83**	**0.88**	**0.74**	**−** **0.35**		**−0.29**
16.	Work engagement	2.42	1.05	0.01	0.09	**0.50**	**0.61**	**0.45**	**0.70**	**0.66**	−**0.27**	−0.12	−0.10	**−** **0.28**	− **0.27**	**−** **0.33**	**0.75**	−**0.32**	

Note: Correlations above the diagonal are person-level correlations in the full sample (*n* = 135). Correlations below the diagonal are person-level correlations in the matched sample (*n* = 101). Correlations in bold type are significant at *p* < 0.05. ^a^ 0 male, 1 female; ^b^ for gender and age *n* = 131 (full sample) and *n* = 99 (matched sample). Bold font indiciates significant correlations.

**Table 3:** Results from Multilevel Analysis Predicting Work Engagement.

Parameter	Model 1			Model 2		
	γ	*SE*	*t*	γ	*SE*	*t*
Level 2 (person-level)						
Intercept	2.44	0.07	33.62	2.43	0.07	33.50
Person-mean positive events	1.21	0.13	9.64 ***	1.21	0.12	9.70 ***
Person-mean negative events	−0.12	0.13	−0.92	−0.13	0.13	−0.97
Level 1 (week-level)						
Time	0.00	0.01	0.33	0.00	0.01	0.43
Positive events (lagged week n−1)	0.03	0.06	0.49	0.03	0.06	0.57
Negative events (lagged week n−1)	0.02	0.08	0.24	−0.02	0.08	−0.20
Positive events (week n)	0.74	0.06	12.52 ***	0.72	0.06	12.12 ***
Negative events (week n)	0.07	0.08	0.86	0.06	0.08	0.77
Interactions						
Positive events x lagged positive events	−0.07	0.11	−0.61	−0.09	0.12	−0.76
Negative events x lagged negative events	−0.02	0.20	−0.07	−0.05	0.20	−0.24
Positive x lagged negative events				0.40	0.15	2.69 **
Negative events x lagged positive				0.10	0.17	0.59
Variance components						
Level 2 intercept variance	0.32			0.33		
Positive events slope variance	0.01			0.02		
Negative events slope variance	0.02			0.01		
Lagged negative events slope variance	0.06			0.06		
Level 1 intercept variance	0.26			0.25		
Deviance (*df*)	920.43		(21)	913.27		* (23)
AIC	962.43			959.27		
BIC	1050.51			1055.74		

Note. *SE* = standard error. *df* = degrees of freedom. * *p* < 0.05. ** *p* < 0.01. *** *p* < 0.001. Deviance = (−2 Residual Log Likelihood). AIC = Akaike information criterion. BIC = Bayesian information criterion.

**Table 4:** Growth Curve Modeling Analysis Predicting Trajectories of Work Engagement Over Time.

Parameter	Growth Model 1			Growth Model 2		
	γ	*SE*	*t*	γ	*SE*	*t*
Level 2 (person-level)						
Intercept	2.48	0.06	38.36	2.47	0.06	38.63
Person-mean positive events	1.11	0.10	11.58 ***	0.98	0.12	8.36 ***
Person-mean negative events	−0.18	0.11	−1.70	−0.11	0.13	−0.91
Level 1 (week-level)						
Time	−0.01	0.01	−2.05 *	−0.01	0.01	−2.20 *
Cross-level interactions						
Person-mean positive events x time				0.03	0.01	1.97 *
Person-mean negative events x time				−0.02	0.02	−1.13
Variance components						
Level 2 intercept variance	0.34			0.33		
Time slope variance	0.00			0.00		
Level 1 intercept variance	0.39			0.39		
Deviance (*df*)	1857.86	*** (8)		1850.93		* (10)
AIC	1873.86			1870.93		
BIC	1911.82			1918.37		

Note: SE = standard error. df = degrees of freedom. * *p* < 0.05. *** *p* < 0.001. Deviance = (−2 Residual Log Likelihood). AIC = Akaike information criterion. BIC = Bayesian information criterion.

**Table 5:** Results from Multilevel Analysis Predicting Work Engagement by Specific Positive and Negative Events Within the Same Week.

Parameter	Model 3		
	γ	*SE*	*t*
Level 1 (week-level)			
Intercept	2.37	0.08	28.38
Time	0.00	0.01	0.46
Goal attainment, problem-solving, task-related success	0.23	0.03	6.70 ***
Perceived competence in or through social interactions	0.07	0.03	1.97 *
Work-related good news	0.05	0.03	1.58
Passively experienced positive events	0.16	0.03	5.59 ***
Praise, appreciation, positive feedback	0.20	0.03	6.45 ***
Technical difficulties, problems with work tools and equipment	0.00	0.03	0.17
Health complaints	−0.01	0.02	−0.30
Private issues	−0.02	0.03	−0.51
Hindrances in goal attainment, obstacles in completing work tasks, overload	−0.04	0.03	−1.52
Problems in interactions with clients or patients	0.03	0.04	0.80
Ambiguity, insecurity, loss of control	0.07	0.03	2.12 *
Conflicts and communication problems	−0.01	0.04	−0.25
Managerial and internal problems, organizational climate	0.03	0.03	0.10
Variance components			
Level 2 intercept variance	0.77		
Time slope variance	0.00		
Level 1 intercept variance	0.27		
Deviance (*df*)	1693.77		(19)
AIC	1731.77		
BIC	1821.91		

Note: *SE* = standard error. *df* = degrees of freedom. * *p* < 0.05. *** *p* < 0.001. Deviance = (−2 Residual Log Likelihood). AIC = Akaike information criterion. BIC = Bayesian information criterion.

**Table 6:** Results from Multilevel Analysis Predicting Work Engagement in Week *n* Controlling for Work Engagement in Week *n* − 1.

Parameter	Model 4		
	γ	*SE*	*t*
Level 2 (person-level)			
Intercept	1.78	0.12	14.75
Person-mean positive events	0.90	0.11	8.16 ***
Person-mean negative events	−0.09	0.10	−0.91
Level 1 (week-level)			
Time	0.00	0.01	0.27
Positive events (lagged week *n* − 1)	−0.19	0.07	−2.85 *
Negative events (lagged week *n* − 1)	−0.05	0.08	−0.60
Positive events (week *n*)	0.73	0.06	12.19 ***
Negative events (week *n*)	0.03	0.08	0.37
Work Engagement (lagged week *n* − 1)	0.27	0.04	6.29 ***
Interactions			
Positive events x lagged positive events	−0.05	0.12	−0.45
Negative events x lagged negative events	0.02	0.21	0.08
Positive x lagged negative events	0.37	0.15	2.43 *
Negative events x lagged positive	0.08	0.18	0.46
Variance components			
Level 2 intercept variance	0.17		
Positive events slope variance	0.01		
Negative events slope variance	0.04		
Lagged negative events slope variance	0.07		
Level 1 intercept variance	0.27		
Deviance (*df*)	889.48		(24)
AIC	937.48		
BIC	1038.14		

Note: *SE* = standard error. *df* = degrees of freedom. * *p* < 0.05. *** *p* < 0.001. Deviance = (−2 Residual Log Likelihood). AIC = Akaike information criterion. BIC = Bayesian information criterion.

## Data Availability

Data supporting reported results can be found at https://doi.org/10.17605/OSF.IO/JTPWV, (accessed on 7 June 2021).

## References

[1] Morgeson F.P., Mitchell T.R., Liu D. (2015). Event system theory: An event-oriented approach to the organizational sciences. Acad. Manag. Rev..

[2] George J.M., Jones G.R. (2000). The role of time in theory and theory building. J. Manag..

[3] Mitchell T.R., James L.R. (2001). Building better theory: Time and the specification of when things happen. Acad. Manag. Rev..

[4] Sonnentag S. (2012). Time in organizational research: Catching up on a long neglected topic in order to improve theory. Organ. Psychol. Rev..

[5] Aguinis H., Bakker R.M. (2021). Time is of the essence: Improving the conceptualization and measurement of time. Hum. Resour. Manag. Rev..

[6] Bakker A.B., Demerouti E. (2017). Job Demands–resources theory: Taking stock and looking forward. J. Occup. Health Psychol..

[7] Ohly S., Schmitt A. (2015). What makes us enthusiastic, angry, feeling at rest or worried? Development and validation of an affective work events taxonomy using concept mapping methodology. J. Bus. Psychol..

[8] Schaufeli W.B., Salanova M., González-Romá V., Bakker A.B. (2002). The measurement of engagement and burnout: A two sample confirmatory factor analytic approach. J. Happiness Stud..

[9] Bakker A.B. (2011). An evidence-based model of work engagement. Curr. Dir. Psychol. Sci..

[10] Christian M.S., Garza A.S., Slaughter J.E. (2011). Work engagement: A quantitative review and test of its relations with task and contextual performance. Pers. Psychol..

[11] Crawford E.R., LePine J.A., Rich B.L. (2010). Linking job demands and resources to employee engagement and burnout: A theoretical extension and meta-analytic test. J. Appl. Psychol..

[12] Bakker A.B. (2014). Daily Fluctuations in Work Engagement: An Overview and Current Directions. Eur. Psychol..

[13] Weiss H.M., Cropanzano R., Staw B.M., Cummings L.L. (1996). Affective Events Theory: A theoretical discussion of the structure, causes and consequences of affective experiences at work. Research in Organizational Behavior: An Annual Series of Analytical Essays and Critical Reviews.

[14] Daniels K. (2006). Rethinking job characteristics in work stress research. Hum. Relat..

[15] Kuba K., Scheibe S. (2017). Let it be and keep on going! Acceptance and daily occupational well-being in relation to negative work events. J. Occup. Health Psychol..

[16] Wickham R.E., Knee C.R. (2013). Examining temporal processes in diary studies. Personal. Soc. Psychol. Bull..

[17] Fuller J.A., Stanton J.M., Fisher G.G., Spitzmüller C., Russell S.S., Smith P.C. (2003). A Lengthy Look at the Daily Grind: Time Series Analysis of Events, Mood, Stress, and Satisfaction. J. Appl. Psychol..

[18] Weiss H.M., Rupp D.E. (2011). Experiencing work: An essay on a person-centric work psychology. Ind. Organ. Psychol. Perspect. Sci. Pract..

[19] Bledow R., Schmitt A., Frese M., Kühnel J. (2011). The affective shift model of work engagement. J. Appl. Psychol..

[20] Gross S., Semmer N.K., Meier L.L., Kälin W., Jacobshagen N., Tschan F. (2011). The effect of positive events at work on after-work fatigue: They matter most in face of adversity. J. Appl. Psychol..

[21] Ilies R., Aw S.S.Y., Pluut H. (2015). Intraindividual models of employee well-being: What have we learned and where do we go from here?. Eur. J. Work. Organ. Psychol..

[22] Meurs J.A., Perrewé P.L. (2011). Cognitive activation theory of stress: An integrative theoretical approach to work stress. J. Manag..

[23] Hobfoll S.E. (1989). Conservation of resources: A new attempt at conceptualizing stress. Am. Psychol..

[24] Bono J.E., Glomb T.M., Shen W., Kim E., Koch A.J. (2013). Building positive resources: Effects of positive events and positive reflection on work stress and health. Acad. Manag. J..

[25] Halbesleben J.R.B., Neveu J.-P., Paustian-Underdahl S.C., Westman M. (2014). Getting to the “COR”: Understanding the role of resources in conservation of resources theory. J. Manag..

[26] Sinclair R.R., Sliter M., Mohr C.D., Sears L.E., Deese M.N., Wright R.R., Cadiz D., Jacobs L. (2015). Bad versus Good, What Matters More on the Treatment Floor? Relationships of Positive and Negative Events with Nurses’ Burnout and Engagement. Res. Nurs. Health.

[27] Wang N., Zhu J., Dormann C., Song Z., Bakker A.B. (2019). The daily motivators: Positive work events, psychological needs satisfaction, and work engagement. Appl. Psychol. Int. Rev..

[28] Beal D.J., Weiss H.M., Barros E., MacDermid S.M. (2005). An episodic process model of affective influences on performance. J. Appl. Psychol..

[29] Demerouti E., Cropanzano R. (2017). The buffering role of sportsmanship on the effects of daily negative events. Eur. J. Work. Organ. Psychol..

[30] Zerubavel E. (1989). The Seven Day Circle: The History and Meaning of the Week.

[31] Dormann C., Van de Ven B., Dollard M.F., Shimazu A., Bin Nordin R., Brough P., Tuckey M.R., Dollard M.F., Shimazu A., Bin Nordin R., Brough P., Tuckey M.R. (2014). Timing in methods for studying psychosocial factors at work. Psychosocial Factors at Work in the Asia Pacific.

[32] Meier L.L., Cho E., Dumani S. (2016). The effect of positive work reflection during leisure time on affective well-being: Results from three diary studies. J. Organiz. Behav..

[33] Ilies R., Keeney J., Goh Z.W. (2015). Capitalising on positive work events by sharing them at home. Appl. Psychol. Int. Rev..

[34] Diener E., Lucas R.E., Scollon C.N. (2006). Beyond the hedonic treadmill: Revising the adaptation theory of well-being. Am. Psychol..

[35] Fredrickson B.L., Branigan C. (2005). Positive emotions broaden the scope of attention and thought-action repertoires. Cogn. Emot..

[36] Llorens S., Schaufeli W.B., Bakker A., Salanova M. (2007). Does a positive gain spiral of resources, efficacy beliefs and engagement exist?. Comput. Hum. Behav..

[37] Solinger O.N., Hofmans J., Bal P.M., Jansen P.G.W. (2016). Bouncing back from psychological contract breach: How commitment recovers over time. J. Organ. Behav..

[38] Gump B.B., Matthews K.A. (1999). Do background stressors influence reactivity to and recovery from acute stressors?^1^. J. Appl. Soc. Psychol..

[39] Farmer A.S., Kashdan T.B. (2015). Stress sensitivity and stress generation in social anxiety disorder: A temporal process approach. J. Abnorm. Psychol..

[40] McEwen B.S. (2007). Physiology and neurobiology of stress and adaptation: Central role of the brain. Physiol. Rev..

[41] Lazarus R.S., Folkman S. (1987). Transactional theory and research on emotions and coping. Eur. J. Personal..

[42] Seery M.D., Leo R.J., Lupien S.P., Kondrak C.L., Almonte J.L. (2013). An upside to adversity? Moderate cumulative lifetime adversity is associated with resilient responses in the face of controlled stressors. Psychol. Sci..

[43] Frese M., Zapf D., Cooper C.L., Payne R. (1988). Methodological issues in the study of work stress: Objective vs subjective measurement af work stress and the question of longitudinal studies. Causes, Coping and Consequences of Stress at Work.

[44] Bolger N., DeLongis A., Kessler R.C., Schilling E.A. (1989). Effects of daily stress on negative mood. J. Personal. Soc. Psychol..

[45] Williams K.J., Suls J., Alliger G.M., Learner S.M., Wan C.K. (1991). Multiple role juggling and daily mood states in working mothers: An experience sampling study. J. Appl. Psychol..

[46] Folkman S. (2008). The case for positive emotions in the stress process. Anxiety Stress Coping: Int. J..

[47] Tsai W.-C., Chen C.-C., Liu H.-L. (2007). Test of a model linking employee positive moods and task performance. J. Appl. Psychol..

[48] Tice D.M., Baumeister R.F., Shmueli D., Muraven M. (2007). Restoring the self: Positive affect helps improve self-regulation following ego depletion. J. Exp. Soc. Psychol..

[49] Grebner S., Elfering A., Semmer N.K., Perrewé P.L., Halbesleben J.R.B., Rose C. (2010). The success resource model of job stress. New Developments in Theoretical and Conceptual Approaches to Job Stress.

[50] Gorgievski M.J., Hobfoll S.E., Halbesleben J.R.B. (2008). Work can burn us out and fire us up. Handbook of Stress and Burnout in Health Care.

[51] Kühnel J., Sonnentag S., Bledow R. (2012). Resources and time pressure as day-level antecedents of work engagement. J. Occup. Organ. Psychol..

[52] MacCallum R.C., Browne M.W. (1993). The use of causal indicators in covariance structure models: Some practical issues. Psychol. Bull..

[53] Schaufeli W.B., Bakker A.B., Salanova M. (2006). The measurement of work engagement with a short questionnaire: A cross-national study. Educ. Psychol. Meas..

[54] Sonnentag S. (2003). Recovery, work engagement and proactive behavior: A new look at the interface between nonwork and work. J. Appl. Psychol..

[55] Geldhof G.J., Preacher K.J., Zyphur M.J. (2014). Reliability estimation in a multilevel confirmatory factor analysis framework. Psychol. Methods.

[56] Huang F.L. Conducting Multilevel Confirmatory Factor Analysis Using R. http://faculty.missouri.edu/huangf/data/mcfa/MCFAinRHUANG.pdf.

[57] Hayes A.F., Coutts J.J. (2020). Use Omega Rather than Cronbach’s Alpha for Estimating Reliability. But…. Commun. Methods Meas..

[58] Rosseel Y. (2012). Lavaan: An R package for structural equation modeling. J. Stat. Softw..

[59] Li C.-H. (2016). Confirmatory factor analysis with ordinal data: Comparing robust maximum likelihood and diagonally weighted least squares. Behav. Res. Methods.

[60] Jorgensen T.D., Pornprasertmanit S., Schoemann A.M., Rosseel Y. (2021). Useful Tools for Structural Equation Modeling [R Package SemTools Version 0.5-4]; Comprehensive R Archive Network (CRAN). https://CRAN.R-project.org/package=semTools.

[61] Raudenbush S.W. (2002). Hierarchical Linear Models: Applications and Data Analysis Methods.

[62] Pinheiro J.C., Bates D.M. (2000). Mixed-Effects Models in S and S-PLUS.

[63] Enders C.K., Tofighi D. (2007). Centering predictor variables in cross-sectional multilevel models: A new look at an old issue. Psychol. Methods.

[64] Paccagnella O. (2006). Centering or not centering in multilevel models? The role of the group mean and the assessment of group effects. Eval. Rev..

[65] West S.G., Ryu E., Kwok O.-M., Cham H. (2011). Multilevel modeling: Current and future applications in personality research. J. Personal..

[66] Chen G., Bliese P.D., Mathieu J.E. (2005). Conceptual framework and statistical procedures for delineating and testing multilevel theories of homology. Organ. Res. Methods.

[67] Kreft I.G.G., de Leeuw J., Aiken L.S. (1995). The effect of different forms of centering in hierarchical linear models. Multivar. Behav. Res..

[68] Bliese P.D., Ployhart R.E. (2002). Growth modeling using random coefficient models: Model building, testing and illustrations. Organ. Res. Methods.

[69] Preacher K.J., Curran P.J., Bauer D.J. (2006). Computational tools for probing interactions in multiple linear regression, multilevel modeling and latent curve analysis. J. Educ. Behav. Stat..

[70] Hülsheger U.R., Lang J.W.B., Depenbrock F., Fehrmann C., Zijlstra F.R.H., Alberts H.J.E.M. (2014). The power of presence: The role of mindfulness at work for daily levels and change trajectories of psychological detachment and sleep quality. J. Appl. Psychol..

[71] Syrek C.J., Weigelt O., Peifer C., Antoni C.H. (2017). Zeigarnik’s sleepless nights: How unfinished tasks at the end of the week impair employee sleep on the weekend through rumination. J. Occup. Health Psychol..

[72] Bakker A.B., Demerouti E. (2007). The job demands-resources model: State of the art. J. Manag. Psychol..

[73] Hoffman E.L., Lord R.G. (2013). A taxonomy of event-level dimensions: Implications for understanding leadership processes, behavior, and performance. Leadersh. Q..

[74] Boswell W.R., Shipp A.J., Payne S.C., Culbertson S.S. (2009). Changes in newcomer job satisfaction over time: Examining the pattern of honeymoons and hangovers. J. Appl. Psychol..

[75] Boswell W.R., Boudreau J.W., Tichy J. (2005). The relationship between employee job change and job satisfaction: The honeymoon-hangover effect. J. Appl. Psychol..

[76] Lee T.W., Mitchell T.R. (1994). An alternative approach: The unfolding model of voluntary employee turnover. Acad. Manag. Rev..

[77] Kacmar K.M., Witt L.A., Zivnuska S., Gully S.M. (2003). The interactive effect of leader-member exchange and communication frequency on performance ratings. J. Appl. Psychol..

[78] Bass B.M., Vecchio R.P. (1997). From transactional to transformational leadership: Learning to share the vision. Leadership: Understanding the Dynamics of Power and Influence in Organizations.

[79] Grant A.M., Fried Y., Juillerat T. (2010). Work matters: Job design in classic and contemporary perspectives. APA Handbook of Industrial and Organizational Psychology, Volume 1: Building and Developing the Organization.

[80] Mitchell T.R., Thompson L., Peterson E., Cronk R. (1997). Temporal adjustments in the evaluation of events: The “rosy view”. J. Exp. Soc. Psychol..

[81] Spector P.E. (2006). Method variance in organizational research: Truth or urban legend?. Organ. Res. Methods.

[82] Casper A., Tremmel S., Sonnentag S. (2019). The power of affect: A three-wave panel study on reciprocal relationships between work events and affect at work. J. Occup. Organ. Psychol..

[83] Dormann C., Griffin M.A. (2015). Optimal Time Lags in Panel Studies. Psychol. Methods.

[84] Driver C.C., Oud J.H.L., Voelkle M.C. (2017). Continuous time structural equation modeling with R package ctsem. J. Stat. Softw..

[85] Bliese P.D., Adler A.B., Flynn P.J. (2017). Transition processes: A review and synthesis integrating methods and theory. Annu. Rev. Organ. Psychol. Organ. Behav..

[86] Koopmann J., Lanaj K., Bono J., Campana K. (2016). Daily shifts in regulatory focus: The influence of work events and implications for employee well-being. J. Organiz. Behav..

[87] French K.A., Allen T.D. (2020). Episodic work-family conflict and strain: A dynamic perspective. J. Appl. Psychol..

[88] Bliese P.D., Lang J.W.B. (2016). Understanding relative and absolute change in discontinuous growth models: Coding alternatives and implications for hypothesis testing. Organ. Res. Methods.

[89] Weigelt O., Syrek C.J., Schmitt A., Urbach T. (2019). Finding peace of mind when there still is so much left undone-a diary study on how job stress, competence need satisfaction, and proactive work behavior contribute to work-related rumination during the weekend. J. Occup. Health Psychol..

[90] Rauvola R.S., Rudolph C.W., Zacher H. Handling time in occupational stress and well-being research: Considerations, examples and recommendations. psyarxiv.com/9bwcd..

